# Seroprevalence of SARS-CoV-2 antibody among individuals aged above 15 years and residing in congregate settings in Dire Dawa city administration, Ethiopia

**DOI:** 10.1186/s41182-021-00347-7

**Published:** 2021-07-10

**Authors:** Tamrat Shaweno, Ibrahim Abdulhamid, Lemlem Bezabih, Daniel Teshome, Behailu Derese, Hiwot Tafesse, Debebe Shaweno

**Affiliations:** 1 Department of Epidemiology, Faculty of Public Health, Jimma University Institute of Health, Jimma, Ethiopia; 2 Dire Dawa Administration Regional Health Bureau, Dire Dawa, Ethiopia; 3 World Health Organization, Addis Ababa, Ethiopia; 4 School of Health and Related Research, University of Sheffield, Sheffield, UK

**Keywords:** Above 15 years, Dire Dawa, Ethiopia, SARS-CoV-2, Seroprevalence

## Abstract

**Background:**

Determining the extent of seropositivity of SARS-CoV-2 antibody has the potential to guide prevention and control efforts. We aimed to determine the seroprevalence of SARS-CoV-2 antibody among individuals aged above15 years and residing in the congregate settings of Dire Dawa city administration, Ethiopia.

**Method:**

We analyzed COVID-19 seroprevalence data on 684 individuals from a community based cross-sectional survey conducted among individuals aged above 15 years and residing in congregate settings in Dire Dawa from June 15 to July 30, 2020. Data were collected using interview and blood sample collection. Participants were asked about demographic characteristics, COVID-19 symptoms, and their practice of preventive measures. Seroprevalence was determined using SARS-CoV-2 IgG test. Bivariate and multivariate multilevel mixed effects logistic regression model was fitted and statistical significance was set at *p* value < 0.05.

**Result:**

The estimated SARS-CoV-2 seroprevalence was 3.2% (95 % CI 2.0–4.8) in the study region with no differences by age and sex but considerable differences were observed by self-reported practice of COVID-19 preventive measures. The cluster effect is not significant (*P* = 0.396) which has suggested no evidence of heterogeneity in SARS-CoV-2 seroprevalence among the clusters. The odds of SARS-CoV-2 antibody seroprevalence were higher for individuals who were employed and work by moving from home to work area (AOR; 9.73 95% CI 2.51, 37.68), reported of not wearing facemasks when leaving home (AOR; 6.4 95% CI 2.30, 17.66) and did not practice physical distancing measures (AOR; 10 95% CI 3.01, 33.20) compared to their counterparts, respectively. Our estimated seroprevalence of SARS-CoV-2 among participants who reported not to have practiced social distancing measures was 12.8 (95% CI, 7.0, 19) and 1.5 (95% CI, 0.5, 2.5) among those who reported of practicing them. More than 80% of study participants reported of implementing infection prevention measures (face masks and physical distancing recommendations).

**Conclusion:**

The detected SARS-CoV-2 seroprevalence among the study participants was low at the time of the survey indicating higher proportion of population yet to be infected. COVID-19 preventive measures were associated with reduced seroprevalence and should be promoted to avoid transmission to the uninfected majority.

## Introduction

Coronavirus disease 2019 (COVID-19) emerged from Wuhan, China, in December 2019 as a novel respiratory illness caused by severe acute respiratory syndrome coronavirus 2 (SARS-CoV-2). Subsequently, it was declared as a pandemic that is a threat for every country in mid-March 2020 by the World Health Organization (WHO) [1]. Since then, the disease has claimed millions of lives and has affected the economy and health throughout the world. The spectrum of SARS-CoV-2 severity varies broadly, from asymptomatic infection to severe complication like organ failure and death [2, 3].

Ethiopia reported the first case in March 2020 and subsequently identified the epicenter at the national capital Addis Ababa; meanwhile, clusters were also detected in the regional capitals including Dire Dawa. As of March 06/2021, there were over 165,000 cases and more than 2000 deaths from COVID-19 in Ethiopia [4]. Although, there was a single serosurvey among asymptomatic people recruited from a clinical laboratory in May 2020 in Addis Ababa, Ethiopia, using only 99 people tested [5], the actual population seroprevalence of disease, and infection have not been measured. Therefore, comprehensive population level studies are required [6–8].

Population level COVID-19 seroprevalence can allow inferences to be made about the extent of infection [9–12]. These findings can have the potential to guide local preventive or control measures [13–16]. Population level seroprevalence studies also provide the magnitude of population who have not yet been infected; a vital information to plan for future transmission control measures and health-care needs. Therefore, this survey was designed to measure the seroprevalence of SARS-CoV-2 antibody among individuals aged above 15 years and residing in congregate settings in Dire Dawa city administration, Ethiopia, which is adjacent to Somali Region that shares a border with Djibouti; where there is high population movement and high-risk social interactions. Data from this survey will serve as a benchmark to show changes in transmission rates in the studied population across time.

## Methods

### Design, setting, and population

This study was part of the nationwide SARS-CoV-2 serosurvey conducted in Ethiopia from mid-June to end of July 2020. The national seroprevalence survey collected data from participants above the age of 15 from all 11 administrative regions of Ethiopia. The sample size of 750 was calculated assuming 7.5% seroprevalence and margin of error of 0.02 at 95% confidence interval. As per the national quota of 16% of households to be included into the survey, 300 households have been included into the survey using systematic random sampling. Nationally allocated quota of 750 sample size was proportionally distributed to these enumeration sites depending on the total number of households available at each enumeration site. We obtained 684 complete samples for Dire Dawa city administration from the Ethiopian Public Health Institute (EPHI). This scale of samples meant that the study can sufficiently reflect the burden at the time of the study. Individuals who resided in households of the selected enumeration areas were included into the study. Individuals with prior history of RT-PCR test or being positive for RT-PCR test were excluded from the study due to the background characteristics of the study participants that might affect the level of participation into the study; which in turn might introduce selection bias.

Dire Dawa is located 515 km from the national capital, Addis Ababa to the east. The current total population of Dire Dawa administration is 507, 000 (323, 000 for Dire Dawa city administration) based on the population projection by the Ethiopian Central Statistical Agency [17]. Dire Dawa administration regional health bureau was among the other sectors to engage early into the establishment of emergency operation centers and task forces following the first documented COVID 19 case in Addis Ababa on March 13, 2020.

SARS-CoV-2 serosurvey was conducted among individuals aged above 15 years and residing in randomly selected households from purposively selected 11 enumeration areas in overcrowded neighborhoods. Overcrowded neighborhoods used in this survey constituted settings with residential houses from 1330 to 8050 within 1 km^2^ area as defined by the Ethiopian Central Statistical Agency. At the time of survey, schools were closed, and social gatherings including religious congregations were restricted in Ethiopia.

### Data collection tool, procedure, and quality control

A sample size of 750 individuals were recruited from the 11 enumeration sites (~70 individuals per enumeration site), and roughly 30 households per enumeration areas were visited and all adults available in hold holds were studied. Households were selected using simple random sampling. Data were collected by trained data collectors on electronic data collection forms using tools adopted from similar other studies [11, 18–21]. Open Data Kit (ODK) was used to collect data electronically. Venous blood samples were collected by trained laboratory technicians, nurses, and medical doctors.

Participants were asked history of symptoms within the last 4 months compatible with COVID-19 (fever, chills, fatigue, myalgia, sore throat, cough, shortness of breath, chest pain, headache, nausea, vomiting, headache, and anosmia). They were also asked about contact with suspected or confirmed cases and their practice of COVID-19 prevention measures (i.e., wearing face masks, social distancing). In this study, proper hand hygiene practice was defined as a person washes hands; the front, back, finger tips, rub thumb, and palms with adequate water and detergent at least for 20–30 s or uses sanitizer/hand rub to the level of compliance before getting in the facility or taking the services. Similarly, proper physical distance was defined as a person keeps 1 m away from another person during getting services, during greetings, during shopping, during discussing, or during praying. In addition, mask wearing practice while leaving home was defined as a person covers the mouth and nose with mask or any type of cloth or handkerchief or tissue.

Blood specimen was kept in an extraction tube filled with extraction buffer (300 μl) at room temperature (15–30°C) for up to 2 h prior to testing. In addition, refrigerator temperature requirement for the SARS-CoV-2 IgG assay was monitored on samples of each day of use based on the manufacturer’s instruction. Specimen shipment, package, and label were conducted in compliance with applicable and available national and international regulations covering the transport of clinical specimens and infectious substances.

### Detection of SARS-CoV-2 IgG antibodies

Serum samples were processed on the Abbott ARCHITECT instrument using the Abbott SARS-CoV-2 IgG assay following manufacturer’s instructions (SARS-CoV-2 IgG for use with ARCHITECT; Abbott Laboratories, Abbott Park, IL, USA) [22]. Although not independently validated, the Abbott IgG test runs on the ARCHITECT platform with the approval of the Ethiopian Food and Drug Administration. The assay is a chemiluminescent micro-particle immunoassay for qualitative detection of IgG in human serum or plasma against the SARS-CoV-2 nucleoprotein. The IgG antibodies to SARS-CoV-2 in each sample were determined by comparing the chemiluminescent relative light unit (RLU) in the samples to the calibrator RLU. Samples were deemed positive if the index values are above the manufacturer’s recommended cut off value of 1.40. Using an index signal cut-off (S/C) threshold of 1.4, the manufacturer reported a sensitivity of 86.4% after 7 days from symptom onset and 100% after 14 days, and a specificity of 99.6%, using RT-PCR as the gold standard. The algorithm has sensitivity of 0 for infections with symptom onset of less than 3 days [11].

### Statistical analysis

We estimated seroprevalence as the proportion of individuals who had a positive result for IgG antibody. As the study participants did not report COVID-19 symptoms, we did not adjust results for sensitivity of the test. Estimates of overall prevalence and the 95% confidence interval have been generated using Stata 14 [23]. Similarly, statistical differences in COVID-19 seroprevalence between two groups were determined using two population proportion test. Fisher-exact test was used for variables with more than two categories. Multilevel mixed effect binary logistic regression model was fitted at 95% CI using multiple pridictors including household clusterring effectson SARS-CoV-2 seroprevalence. Bivariate and multivariate multilevel mixed effect logistic regression analysis was conducted and statistical significance was set at *p* value < 0.05. Variables with a *p* value <0.2 in the bivariate analysis were included in the multiple multilevel mixed effects logistic regression.

## Results

### Socio demographic characteristics of study participants

Data were analyzed for a total of 684 (91.2%) study participants after discarding contaminated samples. Sixty-six (66) samples were not included into this analysis due to grossly hemolyzed specimens 21 (3.8%), information mismatch on request form 14 (2.1%), insufficient specimen volume 18 (2.7%), and missing labels on container 13 (2.0%). Although we designed to exclude individuals with prior history of RT-PCR test or being positive for RT-PCR test from the study, none is found with prior history of RT-PCR test or being positive for RT-PCR test during the data collection time. The mean age of the study participants was 37. 6 (SD=15.6 years). Nearly, half of the participants 362 (52.9%) were in the age category of 15–34 years and 191 (27.9%) were older than 45 years (Table 1).

**Table 1:** Selected socio-demographic characteristics of study participants for SARS-C-oV-2 seroprevalence, Dire Dawa, Ethiopia, 2020

Characteristics	Category	N (%)
Age (*N* = 684)	15–24	180 (26.3)
	25–34	182 (26.6)
	35–44	131(19.2)
	45–90	191(27.9)
Sex (*N* = 684)	Male	307 (44.9)
	Female	377 (55.1)
Occupation (*N* = 684)	Employed	370 (54.1)
	Jobless	314 (45.9)
Education (*N* = 682)	No formal education	65 (9.5)
	Primary education	201 (29.4)
	Secondary education	296 (43.3)
	Technical or higher education	120 (17.5)

### Behavioral and clinical characteristics

When asked about practicing physical distancing recommendations, more than four fifth 558 (81%) of the respondents did report practicing of physical distancing recommendations; of which 344 (60%) did often practice the recommended physical distancing rule (Table 2). Similarly, from the overall respondents, 583 (85.6%) use face mask while leaving home, 582 (85.5%) avoid religious or other social gatherings involving more than four individuals outside of their households/residence, and only 119 (17.6%) did report hand washing practice and use of sanitizers frequently. Concerning main COVID 19 symptoms, all the interviewed respondents did not show any COVID-19-related symptoms including history of fever, chills, fatigue, muscle ache (myalgia), sore throat cough, and known history of contact with anyone with suspected or confirmed COVID-19 patient from the time the first COVID-19 case was documented in Dire Dawa until this survey data was collected**.**

**Table 2:** Selected clinical and behavioral characteristics of study participants for SARS-CoV-2 seroprevalence, Dire Dawa, East Ethiopia, 2020

Behavioral characteristics against COVID-19	Number (%)
Practice physical distancing recommendations (*n* = 681)	558 (81.9)
Often practice physical distancing recommendations (*n* = 558)	344 (60.0)
Wash hand or use sanitizers (*n* = 677)	120(17.7)
Avoid religious or other social gatherings (*n* = 681)	582(85.5)
Use face mask while leaving home (*n* = 681)	583 (85.6)

### Seroprevalence of SARS-CoV-2 antibody

During the study period, the prevalence of SARS-CoV-2 IgG antibody was 3.2%, 95% CI 95% CI 2.0–4.8). The cluster effects is not significant (*P* = 0.396) suggesting  no evidence of heterogeneity in SARS-CoV-2 seroprevalence among the clusters (households). In addition, the log likelihood was the same for both fixed effects model and fitted full model. Our prevalence estimates did not vary by age and sex (Table 3). However, marked differences were observed in the estimated prevalence by self-reported practice of the recommended behavioral characteristics. The prevalence of SARS-CoV-2 among participants who reported not to have practiced social distancing was 8.5 times the prevalence in their counterparts who reported of practicing social distancing. The corresponding estimates were 12.8 (95% CI, 7.0, 19) and was 1.5 (95% CI 0.5, 2.5) with statistically significant difference (*p* < 0.01).

**Table 3:** Analysis of SARS-CoV-2 seroprevalence by selected factors among individuals aged above 15 years and residing in congregate settings of Dire Dawa city administration, Dire Dawa, Ethiopia, 2020

Variables	Categories	Prevalence (95% CI)	*P* value
Age in years	15–24	5.2 (2.0, 8.0)	*p = 0.36*
	25–34	2.2 (0.1, 4.0)	
	35–44	4.0 (0.6, 7.0)	
	45–90	2.2 (0.1,4.0)	
Sex	Male	3.4 (1.0, 5.0)	*p* = 0.96
	Female	3.3 (1.0, 5.0)	
Employment status	Employed	5.4 (3.0, 8.0)	*p* < 0.01
	Not employed	0.9 (0.1, 2.0)	
Use face cover while leaving home	Yes	2.3 (1.0, 4.0)	*p* < 0.001
	No	10.1 (4.1, 16)	
Avoid going to crowds	Yes	2.3 (1.0, 4.0)	*p* < 0.001
	No	10.0 (4.0, 16)	
Practice physical distancing	Yes	1.5 (0.5, 2.5)	*p* < 0.0001
	No	12.8 (7, 19)	
Frequent hand washing/use of sanitizer	Yes	3.0 (2, 4.0)	*p* = 0.25
	No	5.1 (1.0, 9.0)	

Similarly, we observed 4.5 times higher prevalence among individuals who did not wear facemasks, 10.1% (95% CI 4.1, 16)) compared to individuals who reported of wearing them, 2.3% (95% CI 1.0, 4.0) when leaving home. Participation in social gatherings which involve more than 4 people is linked to increased prevalence. In this study, we estimated a prevalence of 10.0% (95% 4.0, 16) among individuals who did not avoid social gatherings which is 4.5 times higher compared to prevalence among individuals who avoided social gathering events. However, we could not detect a statistically significant difference in the prevalence of SARS-CoV-2 by hand washing practice (*p* = 0.25) (Table 3). With regard to employment, the prevalence of COVID 19 among individuals who were employed and work by moving from home to work area was higher. Individuals who were employed and commute between work and home had estimated prevalence of 5.4 (95% CI, 0.03–0.08) compared to individuals who reported no employment 0.1 (95% CI, 0.1–2.1). This observed difference between the two categories of employment status was statistically significant (*p* = 0.001).

### Predictors of SARS-CoV-2 antibody seroprevalence

The following explanatory variables with *p value* less than 0.20 were selected for multivariate logistic regression during bivariate analysis of factors associated with SARS-CoV-2 seroprevalence. These included age, occupation, use of face mask while leaving home, avoiding religious or other social gatherings, and practicing physical distancing recommendation measures. From those five variables entered into the final model, occupation, use of face mask while leaving home, avoiding religious or other social gathering events, and practicing physical distancing recommendation measures showed significant association with SARS-CoV-2 antibody seroprevalence. The odds of SARS-CoV-2 antibody seroprevalence was higher for individuals who were employed and work by moving from home to work area compared to individuals who reported no employment (AOR; 9.73 95% CI 2.51, 37.68). Similarly, odds of SARS-CoV-2 seroprevalence were higher for individuals who did not wear facemasks compared to individuals who reported of wearing them when leaving home (AOR; 6.37 95% CI 2.30, 17.66). Likewise, the odds of SARS-CoV-2 antibody seroprevalence were higher for individuals who did not practice physical distancing measures compared to individuals who did practice it (AOR; 10 95% CI 3.00, 33.20). We did not observe significant statistical association by age category and by participation status in social gatherings that involve more than four people (Table 4).

**Table 4:** Multilevel mixed effects multivariate logistic regression analysis for predictors of SARS-CoV-2 seroprevalence among individuals aged above 15 years in Dire Dawa, Ethiopia, 2020

Variables	Categories	COR, 95% CI	*P* value	AOR, 95% CI
Age in years (*N* = 684)	15–24	1		1
	25–34	2.45 (0.74,8.09)	0.14	2.1 (0.57,8.2)
	35–44	1.04 (0.26, 4.24)	0.95	1.02 (0.22,4.63)
	45–90	1.86 (0.49,7.01)	0.37	1.39 (0.31,6.18)
Occupation	Employed	5.61 (1.64, 19.14)	<0.01	9.73 (2.51, 37.68)^*^
	Jobless	1.00		1.00
Use face mask while leaving home	Yes	1.00		1.00
	No	4.43 (1.84,10.68)	<0.01	6.37 (2.30,17.66)^*^
Avoid religious or other social gatherings	Yes	1.00		1.00
	No	4.34 (1.82, 10.54)	<0.01	1.02 (0.31, 3.46)
Practice physical distancing recommendations	Yes	1.00		1.00
	No	9.43 (3.32,26.78)	<0.01	10.0 (3.00,33.20)^*^

*1.00:* reference*. CI* confidence interval. *COR* crude odds ratio. *AOR* adjusted odds ratio. ^*^P < 0.05

## Discussion

In this survey, the estimated seroprevalence of SARS-CoV-2 is 3.2% among individuals aged above 15 years in the study setting. The large proportion of population (close to 97%) not yet infected at the time of survey meant that promotion of COVID-19 recommended prevention and control measures would be vital to interrupt the continued community transmission [21].

The prevalence in our study was generally consistent with other studies conducted during that time when the epidemic was in its earlier stage. When the study was undertaken, population level measures that include school closure, restrictions on social gatherings, and physical distancing rules were in place in Ethiopia. However, the estimated SARS-CoV-2 antibody by IgG was higher compared to studies conducted in Wuhan [19] and meta-analysis of global pooled seroprevalence [24]; but lower compared to other studies conducted at Addis Ababa [18], Brazil [20], and Iran [21]. The observed differences might be due to differences in the stages of the pandemic at the time of the surveys, with surveys conducted at the earlier stage of the pandemic more likely to report lower prevalence compared to those conducted at the later stage. Another main source of difference is the presence of and level of enforcement of population level government restrictions against COVID-19 to limit transmission.

Consistent with a study from Brazil [20], we could not detect significant difference in IgG prevalence by sex an age category. Although such lack of effect of age could be related to our particular choice of age category, the differences mean that the risk of getting infection might not vary by sex and age. The importance of those variables might be on the progression of and outcome of disease once infection has occurred.

None of the study participant reported symptoms compatible with COVID-19 and contact with a known COVID-19 case. So, the prevalence observed in this study might be caused predominantly by asymptomatic community transmission. This underlies the importance of promoting COVID-19 preventive measures such as wearing face mask and physical distancing to cut the asymptomatic transmission in the population. However, although it may be argued that lack of symptoms compatible COVID-19 is partly related to recall bias, the role of this bias may not be substantial in our study setting. The main reason is that this study was conducted in a city administration, where residents have exposure to information regarding the symptoms and preventive measures through mainstream media as well local means. Besides there were strong restrictions imposed at different stages of the pandemic. These all measures meant that symptoms are less likely to be forgotten. In addition, higher self-reported practice of the recommended COVID-19 prevention measures (more than 80% for most measures) in our study supports our assertion that the study population has good awareness of the disease. On the other hand, study participants might hide their symptoms to avoid inpatient admission for at least 14 days.

In this study, the employment status of the participant was significantly associated with the seroprevalence of SARS-CoV-2 antibody and significantly higher SARS-CoV-2 seroprevalence was observed among individuals who were employed and commute from home to work and back again compared to those working from home or jobless and is consistent with other studies [9, 24, 25]. Employments often require frequent movements and close social interactions and therefore can increase the risk of asymptomatic transmission of SARS-CoV-2.

In our study, self-reported practice of COVID-19 to COVID-19 prevention recommendations has roles in the observed prevalence. In this study, not practicing the recommended physical distancing measures was significantly associated with SARS-CoV-2 seroprevalence. In particular, we observed 10 times higher prevalence among individuals who do not practice the recommended physical distancing measures. Our finding replicates findings from a meta-analysis [26]. However, such effects were not reported in [25, 27, 28] probably related to the epidemic stage and different state of restrictions at the time these studies.

Similarly, not frequently using face mask while leaving homes did show significant association with SARS-CoV-2 seroprevalence. We also observed, 4.5 times higher prevalence among respondents not frequently using face mask while leaving homes compared to those who frequently use it. Consistent with other studies, this study found higher seroprevalence among those who do not frequently use face masks [29]. In Ethiopia and in our study setting, wearing face mask has become mandatory from the early stages of the epidemic.

Although participating in social gathering events did not show a significant association with SARS-CoV-2 seroprevalence; the seroprevalence of SARS-CoV-2 among study participants who did not avoid going to crowds involving more than four people was more than four times higher compared to those who avoided going to crowds involving more than four people. This finding is consistent with a previous study that indicated disproportionately higher prevalence in individuals involved in crowds [20]. However, we failed to detect significant differences in SARS-CoV-2 seroprevalence by hand washing practice.

Our study has several limitations. The performance of the test depends on time since infection, with limited sensitivity for recent infections. At the earlier stage of the infection, individuals may not yet be able produce detectable antibodies. As, a result, it is likely that the estimated prevalence can be underestimated. Individuals with mild or asymptomatic infections tend to have lower IgG responses, so sensitivity of the tests to detect asymptomatic infections is lower, which could lead to underestimating seroprevalence. On the other hand, ELISA tests have shown higher background reactivity in some sub-Saharan African populations, increasing false positive rates [30, 31], which is speculated to be connected to unspecific immune responses from acute malaria. Therefore, future studies need to look into the validity of the test results in an Ethiopian setting.

Although it was possible to adjust for such variations of test performance by time since infection, we could not adjust our estimates to the sensitivity as none of our study participants reported symptoms compatible with COVID-19. Responses regarding COVID-19 recommended preventive measures were subjective self-reports. It is likely that individuals might not always maintain the recommended physical distance and proper mask wearing but can still positively report causing social desirability bias. Moreover, inclusion of only crowded neighbourhoods of the city administration in this survey means that the estimates cannot be representative for the city administration. Therefore, it is essential to note that these findings are true for high-density regions included in this study and should not be generalized to the entire city administration.

## Conclusions

In conclusion, the findings of this study imply that prevalence of COVID-19 was lower, indicating larger proportion of population was yet to be infected. We also found that lower seroprevalence among people with self-reported practice of COVID-19 prevention measures. Therefore, it is essential to promote and strengthen the recommended COVID 19 preventive measures to cut transmissions with in the community.

## Data Availability

All data that support the findings of this study is available from the corresponding author upon request.

## Abbreviations

IRB: Institutional Review Board
SARS-CoV-2: Severe acute respiratory syndrome coronavirus 2
SOP: Standard operating procedures
